# Accidental and Deliberate Self-Poisoning with Medications and Medication Errors among Children in Rural Sri Lanka

**DOI:** 10.1155/2020/9872821

**Published:** 2020-08-03

**Authors:** Kavinda Dayasiri, S. F. Jayamanne, C. Y. Jayasinghe

**Affiliations:** ^1^Base Hospital Mahaoya, Mahaoya, Sri Lanka; ^2^Faculty of Medicine, University of Kelaniya, Kelaniya, Sri Lanka

## Abstract

*Context*. Pharmaceutical products are the leading cause accidental poisoning in middle- and high-income countries. Patterns of poisoning with medicinal drugs change across different geographic regions and over decades owing to variability in prescription practice, sociocultural factors, safe storage of medicines, and free availability of over the counter medications. *Methods*. This multicentre descriptive study was conducted over a seven-year period (February 2007 to January 2014) to assess patterns and trends of medicinal drug-related poisoning among children less than 12 years of age in thirty-six hospitals across rural Sri Lanka. Children with both accidental and deliberate medication poisonings and medication errors were recruited to the study. Data on poisoning events and medication errors were gathered via patient/parent interviews using multistructured questionnaires that assessed demographic factors, first aid measures, location and circumstances of poisoning, clinical management, and complications. In addition, focus group discussions were performed on all children and their families who had deliberate poisoning events and medication errors. *Results and Conclusions*. Among 1621 children presented with acute poisoning over seven years of age, 410 children had acute poisoning with medications. Male children (225, 54.9%) outnumbered female children. Paracetomol (137, 35.6%), salbutamol (55, 14.3%), and chlorpheniramine (35, 9.1%) were the most commonly poisoned medications. Prospective data at Anuradhapura teaching hospital (*n* = 112) revealed that unsafe first aid measures were practiced on 22 (19.6%) children. Although the majority of children remained asymptomatic (61, 54.5%), neurological symptoms (34, 67%) were predominantly seen in symptomatic children. The majority of poisonings took place within home premises (76, 67.9%). There were 16 reports of medication errors (14.2% of acute poisoning events) either due to erroneous administration by caregivers or erroneous issue of medicines by health workers. The current study did not observe mortality following medication poisonings. This study brings to light the burden of medicinal drug-related poisoning morbidity among children in rural Sri Lanka. Potentially, interventions such as community educational initiatives, written safety warnings, increased use of child resistant containers, and enforcement of laws to bring down accidental medication poisonings need to be implemented, and their effectiveness should be evaluated.

## 1. Introduction

Accidental poisoning of children with medicines is a significant cause of preventable morbidity and accounted for approximately 50% of all poisoning-related inquiries to poison information centres in the United States [1]. Nearly 95% of these events are related to accidental self-poisonings, whilst the remaining 5% are due to medication errors [2]. Furthermore, the burden of medication poisoning in children was steadily increasing [3]. Increased risk of toddlers getting accidentally poisoned by medicines is partly related to the development of locomotion and exploratory behaviour in children [4]. However, poor storage and environmental risk factors can significantly increase the risk of accidental medication poisonings in children [5]. Any medicines can be potentially toxic when taken in overdose or by the wrong route. Pain relief medications, vitamins, and diaper and rash products were among the most commonly ingested medications by accidental poisoning in children [1].

Acute poisoning among children is a growing concern among health professionals caring for children. The patterns of poisoning are variable based on sociocultural factors. Kerosene accounted for a significant number of acute poisonings in a study from western India [6]. Medicinal agents were the most common poisoning agents in studies from Middle East [7] and Europe [8]. Ibuprofen was one of the common medicines implicated in paediatric poisoning in Europe [8].

Both self-harm behavior and substance misuse have been implicated in acute poisoning among adolescents [9]. Deliberate and suicidal poisonings have reportedly been associated with high mortality [10]. In contrast to deliberate poisonings, accidental medication poisonings were more commonly seen in toddlers and mortality was low [8]. Safe storage, community education, and enhanced parental supervision are important interventions for prevention of accidental poisonings [11].

Evidence regarding accidental medication poisoning in the paediatric age group in Sri Lanka is limited. A prospective observational study that was conducted more than two decades ago described patient profiles and poisoning characteristics in children [12]. The evidence suggested that toddlers were predominantly affected, whilst deliberate ingestions were exceedingly rare. Drugs for local use, anticonvulsants, and psychiatric drugs were reported as most commonly ingested medication types, whilst skin disinfectants and phenobarbital were the most commonly ingested medications by children. Lack of adult supervision and free availability of medications were the most common predisposing factors. In 13% of children, the medication had been erroneously administered by an adult to cause toxicity, and misidentification of the medication by the adult caregiver was a predisposing factor. It is likely that poisoning patterns have changed with change in prescription practices, sociocultural factors, and increased use and storage of over-the-counter medications at home. More recent studies in Sri Lanka reported the error in prescriptions with potential risk of drug-drug interactions [13], certain dispensing practices by health professionals [14], and dispensing antibiotics without prescriptions [15] as additional risk factors for medication errors (ME).

The current study aims to identify patterns of self-poisoning with medicinal drugs and medication errors among children in rural Sri Lanka.

## 2. Methods

This multicentre study was based on children living in the North Central Province of Sri Lanka in which, predominantly, a rural population resides. The study was hospital-based and recruited all children who were between nine months and 12 years of age and presented either with accidental or deliberate poisoning of pharmaceutical agents. Children presented to both tertiary-care hospitals of the province and thirty-four other regional hospitals were included in the study after confirmation of the event of pharmaceutical poisoning at the hospital emergency department and, subsequently, at the paediatric ward. Children with doubtful poisoning were excluded from the study.

Data were collected over a seven-year period (February 2007 to January 2014) in four major arms: (1) a two-year prospective observational study at Anuradhapura teaching hospital (February 2012 to January 2014) and qualitative study of children with deliberate poisoning and accidental poisoning events caused by caregivers/other people, (2) a one-year prospective observational study at 34 regional hospitals (January 2013–January 2014), (3) a two-year prospective observational study at Polonnaruwa District General Hospital (February 2012–January 2014), and (4) a five-year retrospective observational study at Anuradhapura teaching hospital (February 2007–January 2012). Anuradhapura teaching hospital functioned as the main referral centre of the entire province. Data were collected both prospectively and retrospectively in this centre to better determine trends of medication self-poisonings over a longer period of time in that centre.

Data were collected using a multistructured pretested questionnaire which assessed demographic factors, first aid measures, location and circumstances of poisoning, clinical management, and complications. Mothers were interviewed on the day of admission by the same investigator to minimized recall and observer bias. Fathers or other caregivers were interviewed when the mother was not available.

In addition to collecting quantitative data, focused group discussions were conducted on children and their families when children presented with either deliberate poisoning or poisoning by a caregiver/other person. All focused group discussions were performed by the principle investigator, and key points were recorded as field notes.

The retrospective study collected data from medical records, and only limited demographic and poison-related data which could be considered reliable and auditable based on discharge registers were collected.

All quantitative data were analysed using SPSS version 19.0. All collected data were subjected to independent audit by South Asian Clinical Toxicology research collaboration. Second-order descriptive and interpretative analysis of the content qualitative data was carried out based on field notes to identify patterns. Ethical clearance for the study was granted by ethical review committees, the faculty of medicine, Rajarata University of Sri Lanka and University of Kelaniya. All participant parents/caregivers provided written informed consent.

## 3. Results

Over a seven-year study period, 1621 children presented with either accidental or deliberate poisoning. Fout hundred and ten children (25.3%) had medication poisoning.  Table 1 shows distribution of poisoning with pharmaceutical products across different age groups.

Table 1 shows patterns of variation in age among children who had medication poisonings.

Among children who had pharmaceutical products poisonings, 225 (54.9%) were male children.  Table 2 shows distribution of poisoning with pharmaceutical products across male and female genders.

Table 3 demonstrates the pattern of poisoning with types of medications among children in North Central Province.

Table 4 shows the pattern of commonly ingested medications among children in different arms of the study.

Similar patterns of drug poisoning were observed in each arm of the study. The most common cause for drug poisoning was ingestion of analgesics (35.6%), and it was mostly secondary to paracetomol. The second commonest group accountable for medicinal poisoning was anticonvulsants (14.6%), and clonazepam was observed in majority of cases. Antiasthma medications were the cause for 9.1% of acute drug poisoning, and salbutamol was accountable for the majority in that category. Antihypertensive medicines caused 7.6% of acute drug poisoning, and the most were secondary to ingestion of enalapril, captopril, and nifedipine. Antihistamines were accountable for 7.6% of drug poisoning, and all were secondary to ingestion of chlopheniramine. Antipsychiatric drugs were less commonly seen in all arms of the current study, and only 5.3% of drug poisoning was caused by that group of medicines.

### 3.1. The Prospective Study at Anuradhapura Teaching Hospital

Among 112 children reported with medication poisoning, 67 children (59.8%) belonged to the 3–5 years age category. Twenty-nine children (25.9%) were less than 2 years of age, whilst 13 children (11.7%) were aged 6–10. Three children were reported to have ingested medicinal poisons in the 10–12 years age group. There were 57 (50.9%) male children, and female children accounted for 55 (49.1%). The majority of mothers (80.4%) and fathers (81.2%) had received education at least up to the secondary level. Approximately 50% of fathers were engaged in farming, security services, or manual labour. The majority of mothers were housewives—79 (70.5%). Only two children presented with nonaccidental poisoning.

The majority of children remained asymptomatic (61, 54.5%) following ingestion of medications. However, neurological symptoms were the predominant group symptoms (sleepiness, drowsiness, vertigo, and dizziness) observed in 67% of symptomatic children. Gastrointestinal symptoms (vomiting and abdominal pain) were seen in 36%, whilst 2.3% and 1.8% had cardiovascular and respiratory symptoms.

The location of poisoning included home premises (76, 67.9%) in majority of children: living room (33, 29.5%), bed room (29, 25.9%), and kitchen (11, 9.8%). Unsafe first aid measures were practiced on 22 children (19.6%), and these measures included forceful administration of water (15 cases), coconut milk (3 cases), lime water (91 cases), and cow's milk (1 case) to induce vomiting.

Twenty-three (20.5%) children were brought to the emergency department more than six hours after the time of ingestion. The reasons for delayed presentation included lack of knowledge and parental concern about the need for emergency management (14 cases) and parents not realizing the possibility of poisoning until children became symptomatic (6 cases). Forty-eight children (42.9%) were transferred to the regional tertiary care centre for further observation and management.

Twenty-one children (18.8%) had emission induction measures at the peripheral hospital. One child received intensive care management. Antidotes were required in eight children; all had acute hepatic injury following paracetomol intoxication, and all children received N-acetyl cysteine.

Table 5 shows the effect of long-term medication use by a family member on poisoning with drugs.

Poisoning with a medicinal agent was reported in 44.9% of children of whom at least one family member is on medication for a chronic illness. Among children from families with no one being on long-term medication, only 21.5% had poisoning with medicinal agents. Application of the Chi square test at 95% confidence interval achieved a Chi value of 22.45 with a significance of *p* < 0.001. Thus, the effect of using a long-term medication by a family member on the probability that the child will be poisoned with a medicinal agent was statistically significant.

### 3.2. Accidental Medicine Poisoning by Caregivers/Others

Sixteen children presented the following medication errors, and these included medication errors either by caregivers or the dispensing pharmacy/health practitioner.

Table 6 shows the profile of children who had poisoning by a caregiver/other person.

### 3.3. Intentional Poisoning of Medicines

Two children had presented with deliberate ingestion of pharmaceutical products; however, both of them recovered without developing complications.  Table 7 presents the profile of children with nonaccidental poisoning of medications.

## 4. Discussion

Accidental ingestion of pharmaceutical products poses a serious threat in that certain medications can be fatal if one or more adult doses are ingested by a smaller child. Medication poisonings reportedly are the most common cause of paediatric poisonings accounting for over 60% of accident-related mortality [16]. The outcome of accidental poisoning, although, is rarely fatal [1, 17]. In many instances, only small quantities of the drug are actually ingested, and there may be uncertainty whether it will prove harmless or whether active measures should promptly be instituted [18, 19]. Medications account for approximately 50% of the total reported poisonings in Western countries [2, 20]. A Sri Lankan study had found that medications accounted for 32% of all acute poisonings [21]. We observed a lesser contribution from drugs to overall acute poisonings in children (410 events, 25.3%), and no mortality was seen in two year prospective series. A similar recent study from Nepal observed equal contribution of medications to overall poisoning although mortality rates were higher [22]. Medications were the cause of poisoning in over 50% of children in one study from Pakistan [23] and 36% of children in a study from South India [24].

The most common group of medicines that resulted in poisoning was analgesics (35.6%), and the most common analgesic was paracetomol. Most analgesics are available as over-the-counter medicines in the community, and almost every household uses them to get relief from pain. When these medicines are stored unsafely at home, it can result in poisoning. Notably, ibuprofen was not implicated as a common medication for poisoning among children in current study. This is due to infrequent use of ibuprofen in children due to high prevalence of dengue in which prescription of ibuprofen is often discouraged. Epilepsy, bronchial asthma, and hypertension are commoner chronic illnesses seen in the community, and drugs are usually prescribed on a fortnightly or monthly basis so that they are available as stocks in the house. Also when children see adults taking these drugs on a regular basis, they tend to experience them by tasting. In our study, medicines given for these illnesses accounted for most of the acute poisonings after analgesics. A study in an urban territory of Sri Lanka [6] had most drug poisonings from local applications such as disinfectants (30%), phenobarbital anticonvulsants (22%), and antipsychiatric drugs (19.5%). We observed most poisonings with analgesics followed by nonphenobarbital anticonvulsants and antiasthma medicines. Poisoning from antipsychiatric drugs comprised only a minority (5.3%). This observation is most likely due to the change in prescription practices over decades and less frequent prescriptions for oral phenobarbital. Oral salbutamol is rarely used on a regular basis in children with bronchial asthma as this has been replaced by inhaled salbutamol in many developed countries. However, it was investigators' observation that short courses of oral salbutamol were still being frequently prescribed by many general practitioners on children who had acute mild-to-moderate exacerbations of bronchial asthma. Oral salbutamol was one of most commonly poisoning medications in childhood more than two decades ago [25, 26]. This observation is infrequently documented in recent studies of other South Asian countries although there are reports of salbutamol-induced severe toxicity [27]. In the United Kingdom (UK), methadone which is a long opioid analgesic given for the treatment of opioid dependence and chronic pain had been implicated as the most common cause of drug-related accidental poisoning deaths among toddlers [28]. In another report from the UK, accidental ingestions of benzodiazepines and methadone accounted for the highest number of admissions needing intensive care treatment in the same age group [29]. However, these medications were less frequently observed in the current study, and the most likely reason is less frequent community use of benzodiazepines and morphine as pain relieving/comforting agents.

Neurological manifestations were the most common type of symptoms, and it is consistent with other studies in the literature [30]. Most children in the current study had the poisoning event within their home premises, and this observation has also been reported similarly in previous local [5] and international [1] studies. A direct relationship between the amount consumed and the risk of adverse clinical effects exists for many toxic substances. However, often, it is difficult to assess the exact dosage taken by a child as these ingestions are often unwitnessed and large amount of a liquid toxin can be ingested or spilled over [31] as that can occur following ingestion of pharmaceutical agents in a liquid form. Acute hepatic injury needing N-acetyl cysteine following paracetomol overdose had been the second most common complication. This observation was similarly seen in other studies [32].

The investigators explored the inside stories of children to whom the poisonous substance was served accidentally by another person. It was consistently observed that the poison was served by a person who is well known to the child, and it was the care taker in majority of incidents. All children were physically and emotionally healthy and mature up to their respective chronological ages. The risk factors were mostly caregiver- and poisonous substances-related and were only rarely related to the child himself. As a child matures, he develops the cognitive abilities to avoid harm and danger even if it is ignorantly inflicted by another person. Most children in the current case series were below five years, and the fact makes them more liable to be poisoned as they are yet to develop cognitive maturity to identify and avoid harm by accidental poisoning.

The colour and texture of certain medicinal poisons can attract children. Similarly, it can mislead the caregiver who is less vigilant on poisoning risks. It was a common parental opinion that awareness regarding these risks, proper storage, and vigilance are the key to preventing these poisoning accidents. Caregiver-related risk factors for served poisoning are multiple and more complex. It was the lack of care takers and family support that resulted in less supervision of the child rather than overcrowding. A noteworthy fraction of fathers were away from the family for breadwinning. It led to events where weak and poor grandparents serve “poisonous substances” instead of medicines without knowledge. Most of the rural parents expected the elder siblings to look after the younger siblings when they are working in cultivation fields. The researchers noted several cases where the elder child served poisons instead of medicines in the absence of adult caregivers.

Poor parental education, as well as anxiety, can result in toxicity following overdose of medicines. Inaccurate dosing is the most common reason for having a medication error, whilst incorrect medication and incorrect route of administration are other causes [33]. Furthermore, inaccurate dosing was reportedly the most common cause for medication error-related mortality [34]. Many mothers were worried about the child's illness and freely offered over-the-counter medicines such as paracetomol to control pain, fever, and subsequent febrile convulsions. These anxieties need to be admitted, though simultaneously, with providing accurate advice on doses and harmful effects of commonly used over-the-counter medicines. Specific education is indeed beneficial to those parents who have fears and concerns regarding febrile convulsions.

It was reported that three poisoning events were either secondary to erroneous prescription of medicine by a general practitioner or issuing of drugs by the pharmacy. Medication errors are a serious medical issue which, at times, could be life threatening [35]. The parents trust health-care workers almost 100%, and specially, for parents living in remote areas with transport difficulties and where there is limited access to physicians, at least, getting medicines for the child is of more concern than to look at whether they are correctly prescribed. Therefore, health-care workers hold much responsibility to ensure that the drugs are appropriately prescribed and issued.

This study, although having several methodology-related strengths including appropriate verification and recruitment of cases, a reasonable and representative study sample, both quantitative and qualitative evaluation of children with deliberate poisoning and medication errors, and independent auditing to ensure quality of retrospective data, had several limitations. The investigators had to limit the number of questions for basic demographic data and basic information on poisons in the retrospective series in order obtain clear and most accurate data. Important information such as complications, clinical features, and outcomes were not collected in the retrospective study as few data were missing or incomplete and could not be auditable based on discharge registers. Whilst limited data in one arm of the study could limit the overall interpretation of poisoning patterns, the investigators ensured that only limited, yet accurate and reliable data, are considered in analysis of retrospective data and comparisons with other arms of the study.

In order to prevent drug poisoning in children, interventions such as improving parental education and promoting doctor-patient communication are likely to be effective [36]. Improving labelling standards by adding stronger warnings about the risk of overdose and provision of weight-based dosing charts for parents are helpful in understanding dosage instructions by less educated caregivers. Engineering modifications to improve safe packing of medicines and enforcement of law have enabled decreasing the number of accidental self-poisonings in children [37]. This study brings to light that both accidental self-poisoning of pharmaceutical products and medication errors are important causes of preventable morbidity in rural Sri Lanka. Potential interventions such as community educational initiatives, written safety warnings, increased use of child-resistant containers, and enforcement of law to bring down accidental medication poisonings need to be implemented, and their effectiveness should be evaluated.

## 5. Conclusions

Acute self-poisoning of pharmaceutical products in children and medication errors by caregivers are significant, yet less widely spoken, health problems in rural Sri Lanka. Paracetomol, salbutamol, and chlorpheniramine were the most commonly reported medications implicated in poisoning. Patterns of poisoning and mortality are different compared to previous local studies and are likely due to changes in prescribing practices, changes in sociocultural risk factors, and free availability and storage of over-the-counter medicines at home. Potential interventions such as community educational initiatives, written safety warnings, increased use of child-resistant containers, and enforcement of law to bring down accidental medication poisonings need to be implemented, and their effectiveness should further be evaluated.

## Figures and Tables

**Table 1:** Patterns of variation in age among children who had medication poisonings (THA- Teaching Hospital Anuradhapura, PGH- Polonnaruwa District General Hospital, and RDHS- Regional Director of Health Services).

Age group	THA retrospective study (*n* − 135) (%)	THA prospective study (*n* − 112) (%)	PDGH (*n* − 108) (%)	RDHS study (*n* − 55) (%)	Total (*n* − 410) (%)
0–2 years	37 (27.4)	29 (25.9)	42 (38.9)	9 (16.4)	117 (28.5)
3–5 years	78 (57.8)	67 (59.8)	50 (46.3)	38 (69)	233 (56.8)
6–10 years	12 (8.9)	13 (11.7)	16 (14.8)	2 (3.6)	43 (10.5)
10–12 years	8 (5.9)	3 (2.7)	—	6 (10.9)	17 (4.2)

**Table 2:** Patterns of variation in sex among children who had medication poisonings (THA- Teaching Hospital Anuradhapura, PDGH- Polonnaruwa District General Hospital, and RDHS- Regional Director of Health Services).

Gender	THA retrospective study (*n* − 135) (%)	THA prospective study (*n* − 112) (%)	PDGH (*n* − 108) (%)	RDHS study (*n* − 55) (%)	Total (*n* − 410) (%)
Male	77 (57)	57 (50.9)	65 (60.2)	26 (47.3)	225 (54.9)
Female	58 (43)	55 (49.1)	43 (39.8)	29 (52.7)	185 (45.1)

**Table 3:** Pattern of poisoning with types of medications among children in North Central Province (THA- Teaching Hospital Anuradhapura, PDGH- Polonnaruwa District General Hospital, and RDHS- Regional Director of Health Services).

Type of medication	THA retrospective study (*n* − 135) (%)	THA prospective study (%)	PDGH (*n* − 108) (%)	RDHS study (*n* − 55) (%)	Total (%)
1. Analgesics	48 (35.5)	39 (33.9)	27 (29)	22 (53.6)	137 (35.6)
2. Anticonvulsants	20 (14.8)	20 (17.3)	9 (9.6)	6 (14.6)	55 (14.3)
3. Antiasthma drugs	14 (10.3)	7 (6.1)	13 (13.9)	2 (4.8)	35 (9.1)
4. Antihypertensive drugs	9 (6.6)	11 (9.5)	6 (6.4)	3 (7.2)	29 (7.6)
5. Antihistamines	9 (6.6)	9 (7.8)	9 (9.6)	2 (4.8)	29 (7.6)
6. Antibiotics	6 (4.4)	9 (7.8)	6 (6.4)	(0)	21 (5.4)
7. Antipsychiatric drugs	4 (3)	9 (7.8)	3 (3.2)	4 (9.6)	20 (5.3)
8. Disinfectants	4 (3)	3 (2.6)	3 (3.2)	1 (2.4)	11 (2.9)
9. Other	21 (15.5)	8 (6.9)	17 (18.2)	1 (2.4)	47 (11.2)

**Table 4:** Patterns of commonly ingested medications among children in North Central Province (THA- Teaching Hospital Anuradhapura, PDGH- Polonnaruwa District General Hospital, and RDHS- Regional Director of Health Services).

Medication	THA retrospective study (*n* − 135) (%)	THA prospective study (%)	PDGH (*n* − 108) (%)	RDHS study (*n* − 55) (%)	Total (%)
1. Paracetomol	48 (35.5)	39 (33.9)	27 (29)	22 (53.6)	137 (35.6)
2. Salbutamol	20 (14.8)	20 (17.3)	9 (9.6)	6 (14.6)	55 (14.3)
3. Chlorpheniramine	14 (10.3)	7 (6.1)	13 (13.9)	2 (4.8)	35 (9.1)

**Table 5:** Relationship between medicinal poisoning and a family member using long-term medication.

	With long-term medication	Without long-term medication
Drug poisoning	**57**	**55**
No drug poisoning	70	201

**Table 6:** Profile of children who had poisoning by a caregiver/other person.

Case No.	Age	Sex	Medication	Circumstance of poisoning
1	9/12	F	Cefuroxime	Mistakenly given by her mother who was educated only up to grade five at school. She gave coconut milk to the child to induce vomiting, and fortunately, it was not aspirated. Her 20-year-old husband was employed as a driver and visited them monthly. They had not safely stored medicines and household chemicals at home
2	1	F	Methyl salicylate	Mistakenly given instead of cough syrup by her father. They had a safe storage facility at home for medicinal drugs, but poor attention was the immediate reason. Storing of the child's medicines along with those of other family members resulted in accidental poisoning
3	3½	F	Camphor oil	Mistakenly given instead of cough syrup by the grandmother. The mother was busy with her job during daytime, and the grandmother helped her in looking after the child. They had kept both camphor oil and the child's medication bottles in the same shelf of the rack
4	3	F	Paracetomol	Given 5 times the normal dose of paracetomol syrup by her 10-year-old sister. Both parents had been busy with agricultural work during daytime, and the elder child took care of her younger sibling at home
5	2	M	Paracetomol	Given three times the normal dose for 24 hours by her mother during a febrile illness before seeking medical advice. They had poor knowledge and concern on toxic effects of higher doses and worried more about fever and subsequent risk of developing febrile convulsions
6	3½	F	Nifedipine	Erroneously issued by a local dispensary
7	9/12	F	Clonazepam	Given instead of her cough medication mistakenly by the mother. Her five-year-old brother was on clonazepam for epilepsy, and both medicines were stored together in the same bottle
8	3	M	Lindane	Given, an organochloride scabicide to drink after dissolving in water, by the mother instead of local application. The child developed 3 convulsions before admission. The mother was not aware of to how to give the drugs though the instructions were appropriately written in English (not in local language)
9	2½	M	Paracetomol	Given by the mother following a viral fever. She was anxious and scared that the child will develop a febrile convulsion. The dose of paracetomol was decided by the mother herself
10	7	M	Paracetomol	Given three times the weight appropriate dose of paracetomol (500 mg 6 hourly) mistakenly by the mother. The mother had not sought medical advice before prescription
11	2	M	Paracetomol	Given two times the weight appropriate dose for five days after it being issued by a local OPD (out patients' department) pharmacy. The mother was not aware that she was giving a higher dose of paracetomol to her child
12	6/12	F	Salbutamol	Given accidentally to drink by her 6-year-old elder sister. The medicine had been kept in the living room with easy access to the elder child, and parents had not been around the children at the time the incident took place. The mother was working during daytime, and all types of poisons (household, medicinal, and pesticides) were poorly stored at home
13	9	M	Paracetomol	Given five times the weight appropriate dose for two days by the mother for viral fever. She did not have enough knowledge on deciding quantities based on weight. She had given paracetomol to him similarly several times previously
14	1	M	Camphor oil	Given instead of cough syrup at home by the grandmother. Both parents were employed and were not available at home during daytime. They have stored medicines and household chemicals together with both being poorly stored
15	1½	M	Haloperidol	Given instead of cough syrup after it was erroneously issued by the local pharmacy. Parents did not notice the error until they sought further medical care for new onset drowsiness
16	½	M	Kerosene oil	Mistakenly given for abdominal pain by the grandmother instead of asamodagam (a traditional medicine). They had kept the “asamodagam” bottle in the same rack with kerosene oil

**Table 7:** Profile of intentional poisoning cases.

	Age, yrs.	Sex	Poison	Circumstance	Background as revealed in FGD
1	11	M	Nifedipine	Self-poisoning	Child with poorly controlled schizophrenia
2	10	M	Tramadol	Self-poisoning	Stress related to academic achievements and verbal abuse by the mother

## Data Availability

Data can be made available on a reasonable request by readers.
